# Supplementary material for: l-Lactic Acid Production Using Engineered Saccharomyces cerevisiae with Improved Organic Acid Tolerance
Source: J Fungi (Basel). 2021 Oct 31;7(11):928. doi: 10.3390/jof7110928 (PMC8624227; doi:10.3390/jof7110928)
Supplement: Supplementary file 1 [file jof-07-00928-s001.zip › jof-1424623-supplementary.pdf]

Journal of
Fungi

MDPI

Supplementary Material

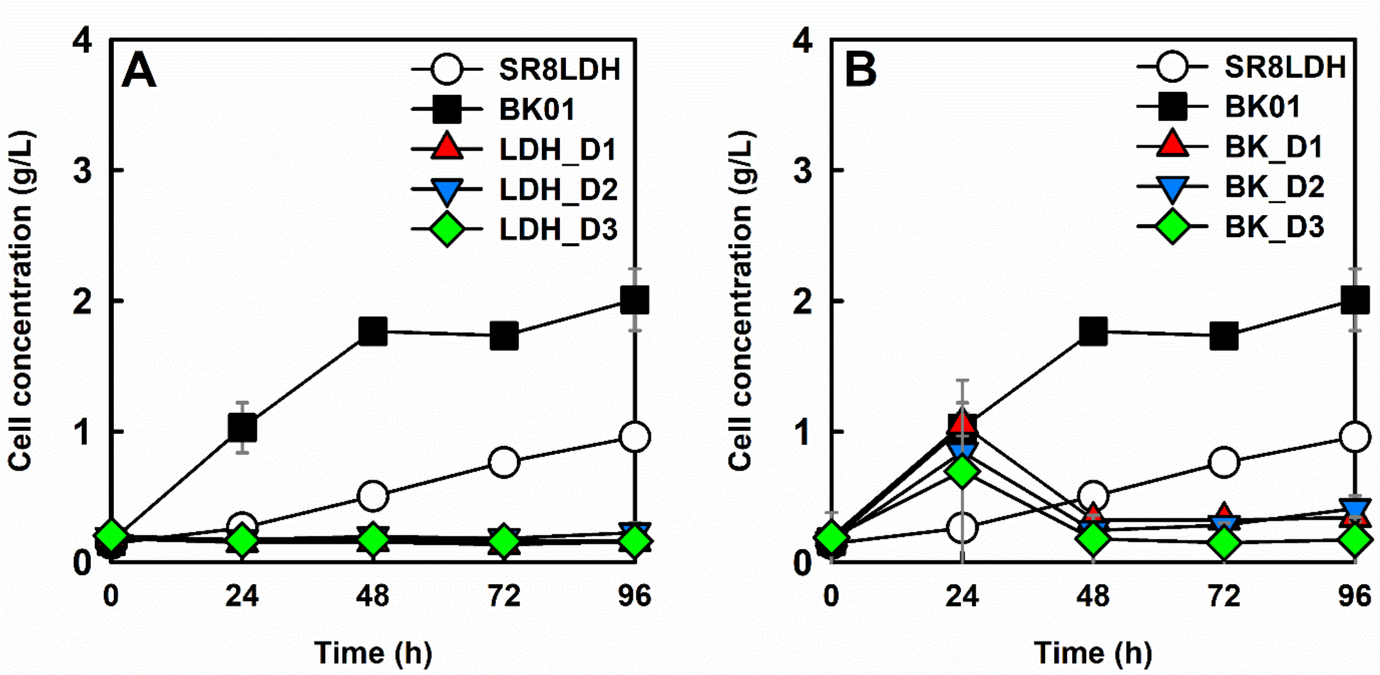

Figure S1. Comparison of volumetric growth rates (g/L) of the reverse engineered strains from SR8 LDH and BK01 in complex medium containing 20 g/L glucose and 8% lactic acid. (**A**) Deletion mutants from SR8LDH; (**B**) deletion mutants from BK01. All experiments were conducted under oxygen-limited conditions (80 rpm), with an initial cell concentration of 0.05 g DCW/L.

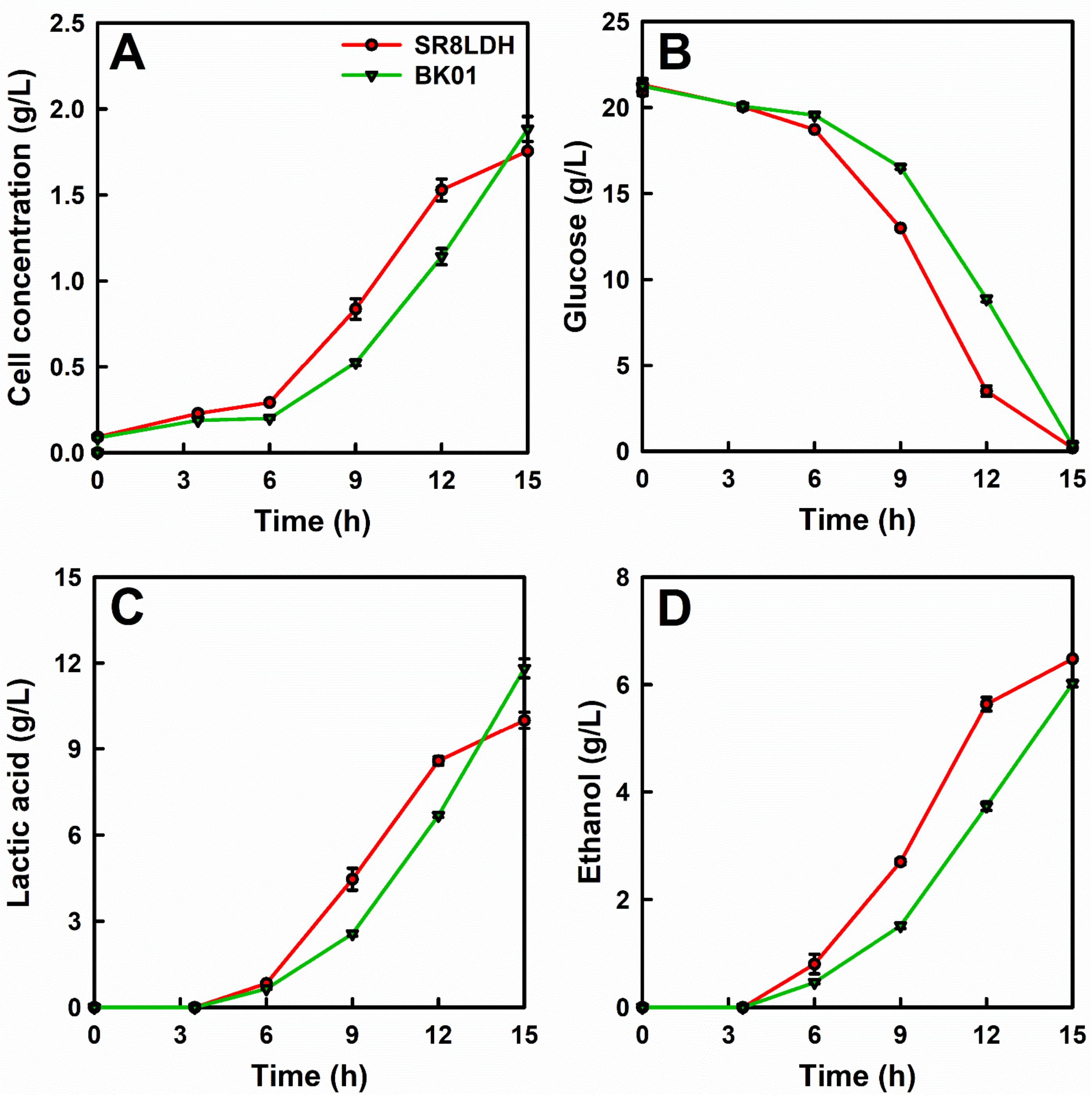

Figure S2. Fermentation profiles of the SR8LDH and BK01 strain in complex medium containing 20 g/L glucose. (**A**) Cell concentration, (**B**) glucose consumption, (**C**) lactic acid production, and (**D**) ethanol production. The values are the mean of three independent experiments, and the error bars indicate the standard deviations. All experiments were conducted under oxygen-limited conditions (80 rpm), with an initial cell concentration of 0.05 g DCW/L.

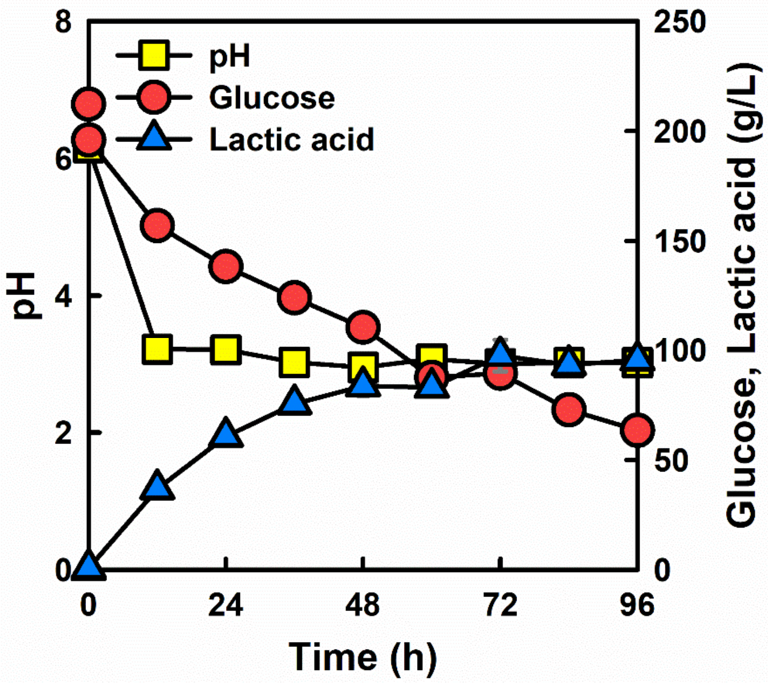

Figure S3. Fermentation profiles of the BK01 strain in complex medium containing 200 g/L glucose under oxygen limited conditions (80 rpm) with initial cell concentration of 10 g DCW/L (g dry cell weight/L).

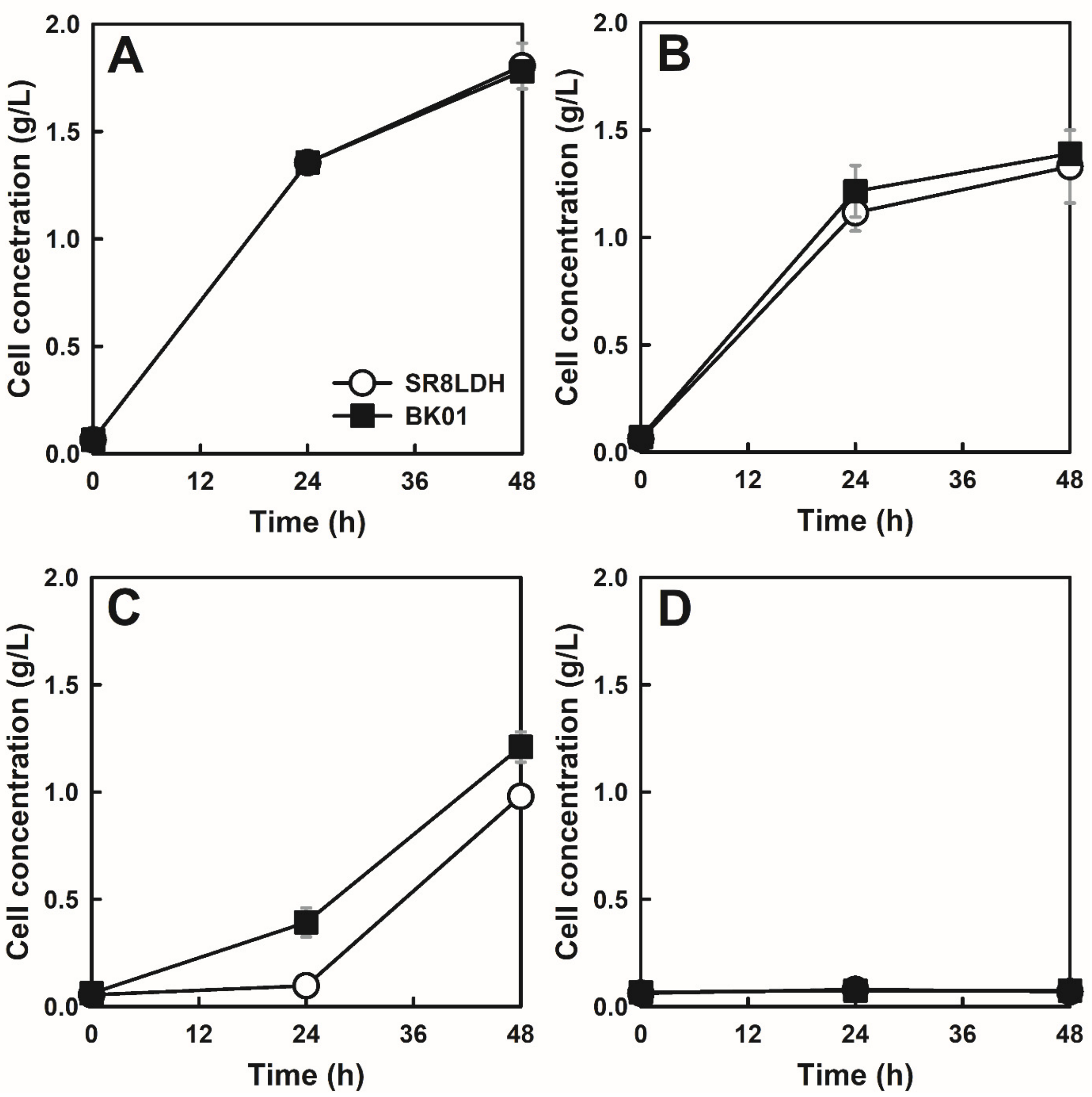

Figure S4. Comparison of volumetric growth rates (g/L-h) of the SR8 LDH and BK01 strains in complex medium containing 20 g/L of glucose and acetic acid. (**A**) 2 g/L, (**B**) 3 g/L, (**C**) 4 g/L, and (**D**) 5 g/L acetic acid is contained in complex medium. All experiments performed under oxygen limited conditions with cell concentration of 0.05 g DCW/L (g dry cell weight/L).

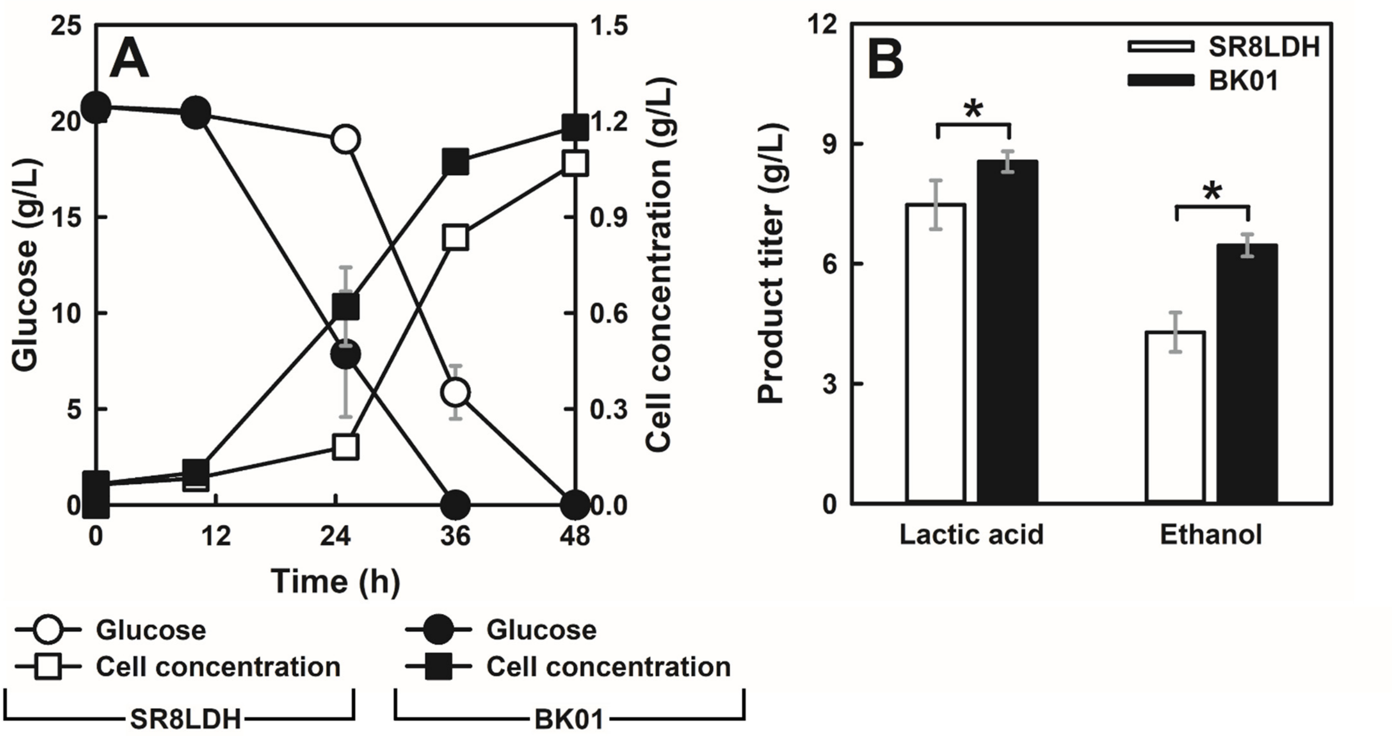

Figure S5. Fermentation profiles of the SR8LDH and BK01 strains in complex medium containing 20 g/L glucose and 4 g/L acetic acid. (**A**) Glucose consumption and cell concentration, (**B**) maximum product titers at 36 h. The values are the mean of three independent experiments, and the error bars indicate the standard deviations. All experiments were conducted under oxygen limited conditions (80 rpm), with an initial cell concentration of 0.05 g DCW/L. Asterisks denote statistically significant differences (Student's t-test, $p < 0.05$).

Table S1. Unique, non-synonymous SNPs in the parental and evolved strain

No	Chr	ORF	Gene	SNP detection by Illumina	Sanger confirmation
1	I	*YAL063C*	*FLO9*	1825 A>T	-
2	II	*YBR112C*	*CYC8*	1611 T>A	-
3	III	*YCR018C*	*SRD1*	289 G>A	PCR failure
4	IV	*YDL248W*	*COS7*	334 G>A	-
				350_351 CT>TG	-
				355 C>A	-
				427 A>T	-
				541 C>T	-
				820 C>T	-
				884 T>C	-
5	IV	*YDL065C*	*PEX19*	1024 C>A	-
6	IV	*YDR150W*	*NUM1*	2683 T>C	Tandom repeats
7	IV	*YDR544C*	-	161 G>C	Sequencing error
				167 G>C	Sequencing error
				173 G>C	Sequencing error
				179 G>C	Sequencing error
8	IX	*YIL169C*	*CSS1*	294_295 CT>AG	PCR failure
				565 G>A	PCR failure
				597_598 AG>TA	PCR failure
				1347_1348 AC>GT	PCR failure
				1363_1364 AC>GA	PCR failure
9	IX	*YIR019C*	*FLO11*	1699 A>C	Tandom repeats
				2090 G>C	Tandom repeats
				2180 G>C	Tandom repeats
10	VII	*YGR023W*	*MTL1*	722 T>C	-
11	VII	*YGR295C*	*COS6*	222 G>A	-
				253 G>A	-
				259 G>C	-
				265 G>T	-
12	VIII	*YHR030C*	*SLT2*	1142_1143 insertion ACA	-
				1145_1146 insertion ACA	-
13	VIII	*YHR056C*	*RSC30*	2014 A>G	-
14	VIII	*YHR143W*	*DSE2*	605_613 deletion CTTCTTCTT	-
15	VIII	*YHR219W*	-	455 T>C	-
16	X	*YJR151C*	*DAN4*	2746 A>G	-
17	XI	*YKL164C*	*PIR1*	297 A>T	-
				302_303 AA>CT	-
				305 C>T	-
				310 G>C	-
18	XII	*YLL066C*	-	2090 T>C	-
				2054 T>C	-
19	XII	*YLL021W*	*SPA2*	2760_2761 GA>AG	-
				2767 G>A	-
				2777 T>C	-
20	XII	*YLR286C*	*CTS1*	1430_1444 deletion GTAGTACAAGCTCAG	-
21	XII	*YLR410W -B*	-	2213 C>T	-
**22**	**XIII**	***YML001W***	***YPT7***	**553 G>A**	**Confirmed**
**23**	**XV**	***YOL159C-A***	**-**	**172 C>A**	**Confirmed**
24	XV	*YOL155C*	*HPF1*	493 A>T	-
				506 A>C	-

Table S2. Guide RNA structure

Description	Sequence
SNR52 promoter	TCTTTGAAAAGATAATGTATGATTATGCTTTCACTCATATTTATACAGAAACTT-<br>GATGTTTTCTTTCGAGTATATACAAGGTGATTACATGTACGTTTGAAGTACAACTCTA<br>GATTTTGTAGTGCCCTCTTGGGCTAGCGGTAAAGGTGCG-<br>CATTTTTTCACACCCTACAATGTTCTGTTCAAAAGATTTTGGTCAAACGCTGTAGAA<br>GTGAAAGTTGGTGCGCATGTTTCGGCGTTCGAAACTTCTCCGCAGTGAAAGA-<br>TAAATGATC
Target sequences	
YPT7.2	ATCATTATAGTCATCTTCAA
YOL159C-A.3	ACTGTGAACTCGATTTATCA
Δ*yol159c-a.1*	GTACACCTACCCGTCACCGG
Structural crRNA	GTTTTAGAGCTAGAAATAGCAAGTTAAAATAAGGCTAGTCCGTTATCAACTT-<br>GAAAAAGTGGCACCGAGTCGGTGGTGC
SUP4 terminator	TTTTTTTGTTTTTTATGTCT

Table S3. Primers

Name	Description	Sequence
For donor DNA preparation for Cas9 engineering		
Kim637	m*YPT7*_F	AACGCCATAAACGTTGATACCGCATTTGAAGAAATTGCAAGGAG-TGCTTTACAACAGAATCAAGCTGATA CAAAAGCTTTTGAAGATGAC
Kim638	m*YPT7*_R	GAAGATACAATTAAGTAGTACAGCTCAACAGCTACAA-GAATTATTTTCTCCATCTAGGCGAATATTGATG GCATCATTATAGTCATCTTC
Kim679	m*YOL159C-A*_F	CTGCTTAAAGCGTAGAGTTC
Kim680	m*YOL159C-A*_R	CTCCAGTCGAGATACGATAC
Kim770	Δ*ypt7*_F	GAATAAGCTCATCCAGTCCAC-TTCTTATCCATATAGAAACCCCTTCTG-TATCAATTCAAATTAAGTGACT GTGAACTCGATTTATCAGGG
Kim771	Δ*ypt7*_R	AAATTATTAATTTTTTTAAGGATACGCTATAAAGGATTACATAA-TAGAAGATACAATTAAGTAGTACAGC CCCTGATAAATCGAGTTCAC
Kim684	Δ*yol159c-a*_F	ATAAGTACTATATATACGCTGCTAGTTTCTTTGTTTTGTCGAGTTTGG-GACCAAACCAATACCATTTGTG GAATCACTGCACATGTACAC
Kim684	Δ*yol159c-a*_R	GTAAGTTTACTGAGTCGACAACAGAGCTAGCAT-ACGCATGAATCTCGGTCTGAcctCCGGTGACGGGTAG GTG-TACATGTGCAGTGATTC
For sequencing confirmation of Cas9 engineering		
Kim569	*YPT7*_F	AAGCAGTCGTTACAGAGGAACAG
Kim570	*YPT7*_R	AAAGTTCGGAACGCATACAC
Kim679	*YOL159C-A*_F	CTGCTTAAAGCGTAGAGTTC
Kim680	*YOL159C-A*_R	CTCCAGTCGAGATACGATAC

Table S4. List of identified intracellular metabolites in SR8LDH and BK01

No.	Metabolite name	Retention time (min)	Average (Peak height)		Standard deviation (Peak height)		P-value	Fold-change
			SR8LDH	BK01	SR8LDH	BK01		
1	Galactonic acid	11.52	3159.83	5113.31	375.99	730.28	0.00	1.62
2	Inositol-4-monophosphate	14.10	21732.80	34975.02	5859.23	6685.40	0.00	1.61
3	Glucose-6-phosphate	13.51	17166.41	25448.42	3408.25	6555.88	0.02	1.48
4	Glutathione	12.69	18613.83	25816.39	1597.19	4945.13	0.01	1.39
5	Maltose	15.78	722539.37	934142.55	58749.37	91535.86	0.00	1.29
6	Galactose	10.81	887478.30	1126514.21	101369.02	147460.97	0.01	1.27
7	Glucose	10.97	650081.18	821610.70	84817.83	105729.50	0.01	1.26
8	Hydroxylamine	4.18	1707.76	2111.38	280.12	307.94	0.04	1.24
9	Leucine	5.76	802136.40	962540.06	44678.38	94589.39	0.00	1.20
10	Ethanolamine	5.75	799349.92	958225.85	44225.52	95076.02	0.00	1.20
11	Methylphosphate	4.83	2633.35	1952.75	307.12	185.23	0.00	-1.35
12	3-Phosphoglycerate	10.18	13890.24	10086.34	1407.66	2596.32	0.01	-1.38
13	Uridine	14.36	42067.14	30308.65	6697.43	4846.25	0.01	-1.39
14	Palmitoleic acid	11.78	44625.57	31809.04	7283.24	1735.48	0.00	-1.40
15	Malate	7.68	13091.78	9197.55	1618.43	2673.80	0.01	-1.42
16	Glycine	6.08	486113.47	336038.90	89401.35	26766.88	0.00	-1.45
17	Fructose-6-phosphate	13.41	10802.92	7164.25	1659.13	959.01	0.00	-1.51
18	UDP-N-acetylglucosamine	10.40	3235.75	2075.16	384.04	280.93	0.00	-1.56
19	Threitol	7.79	3490.23	2216.67	234.93	224.43	0.00	-1.57
20	Threonine	6.81	63817.49	32185.79	6042.87	4621.75	0.00	-1.98
21	Citric acid	10.26	44861.83	15176.83	9059.16	6098.41	0.00	-2.96
22	Galactinol	16.68	33903.50	10454.76	4446.48	1290.92	0.00	-3.24
23	Pyrophosphate	9.12	27142.39	4158.59	16507.64	2697.25	0.01	-6.53
24	N-acetylglutamate	10.08	6233.54	848.82	1508.54	226.20	0.00	-7.34

* Metabolites sorted by fold-change value
* The colored metabolites indicate the five metabolites with the highest (red) and the lowest (blue) fold-change value among significantly different metabolites between SR8LDH and BK01.
